# Estimating meaningful change for The Impact of Weight on Self-Perception (IW-SP) questionnaire among people with type 2 diabetes

**DOI:** 10.1007/s11136-023-03476-0

**Published:** 2023-07-25

**Authors:** Heather L. Gelhorn, Stephen Maher, Helene Sapin, Jiat Ling Poon, Kristina Boye

**Affiliations:** 1 Evidera, Bethesda, MD USA; 2 Eli Lilly and Company, Indianapolis, IN USA; 3 Evidera, 7101 Wisconsin Blvd., Suite 1400, Bethesda, MD 20814 USA

**Keywords:** Meaningful change, IW-SP, Type 2 diabetes, Patient-reported outcome measure, Impact of weight, Self-perception

## Abstract

**Purpose:**

The Impact of Weight on Self-perception Questionnaire (IW-SP) is a three-item patient-reported outcome measure (PROM) instrument assessing the impact of body weight on self-perception. To date no published threshold for meaningful change exists. The objective of this study was to estimate the minimal important change (MIC) for the IW-SP among people with type 2 diabetes.

**Methods:**

Responder analyses were conducted using anchor- and distribution-based approaches with existing clinical trial data (SURPASS-2). As SURPASS-2 did not include a priori anchors, a set of alternative exploratory anchors were identified based on the MICs and items from two conceptually related measures used in the trial as well as percent change in body weight. Exploratory anchors with change estimates that were sufficiently related to change in IW-SP (*r* ≥ 0.30) and were not redundant with other anchors were retained for the MIC analyses. The analyses were conducted in two stages (estimation = 2/3 of sample) to derive initial IW-SP MIC estimates, and a subsequent confirmation stage (remaining 1/3 of sample).

**Results:**

While the most conceptually related anchors and items performed best in responsiveness analyses, all anchors resulted in a similar estimate of minimal meaningful change for the IW-SP total score: a *1-point change* in raw units (1–5-point scale), corresponding to a *25-point change* for transformed scores (0–100 scale). Distribution-based analyses supported these MIC estimates. Results were similar across both stages for all analyses.

**Conclusion:**

The MIC for the IW-SP for patients with T2D is a 25-point change on the transformed score.

**Supplementary Information:**

The online version contains supplementary material available at 10.1007/s11136-023-03476-0.

## Introduction

The association between obesity and diabetes is well established [1–3], and approximately 90% of patients with type 2 diabetes (T2D) are living with obesity or overweight [4]. Weight management is a part of diabetes control, and some diabetes medications result in weight loss. Self-perception related to body weight is an important concept for T2D as it may be associated with patients’ motivation to adhere to the medications and support efforts to reach and maintain a healthy body weight.

The Impact of Weight on Self-perception (IW-SP) [5, 6] is a patient-reported outcome measure (PROM) that assesses self-perception associated with one’s body weight. This measure has been used in clinical trials, however to our knowledge, a minimal important change (MIC) [5–7] has not been established to guide the interpretation of score changes. The MIC refers to the minimal meaningful within-patient group mean change over time [8]. We will use MIC, but this and related concepts are also referred to by other names, including minimally important difference (MID) and minimal clinically important change (MCIC), and simply meaningful change. The MIC is crucial to interpreting whether changes in self-perception related to weight loss, as measured by the IW-SP, represent changes that are meaningful to patients.

The primary objective of this work was to derive an MIC for improvement in the IW-SP total score among individuals with T2D using data from the SURPASS-2 trial [9]. The current investigation was focused on improvements in the IW-SP, since the majority of participants in the clinical trial dataset lost weight.

## Methods

### Measures

The IW-SP, and two other patient-reported outcomes (PROs) instruments were used in this study to help determine the MIC of the IW-SP.

#### Impact of Weight on Self-perception Questionnaire (IW-SP)

The IW-SP contains three items [6]:

1. How often do you feel unhappy with your appearance due to your weight?
2. When going out in public, how often do you feel self-conscious due to your weight?
3. When comparing yourself to others, how often do you feel unhappy, due to your weight?

Each item is rated on a five-point scale: 1 = “always,” 2 = “frequently,” 3 = “sometimes,” 4 = “rarely,” and 5 = “never.” The IW-SP total scores are derived by summing the item scores and dividing by the number of items. The score can be transformed to a range from 0 to 100. Higher IW-SP scores correspond to better self-perception, and the items apply to the present [6].

#### Ability to perform physical activities of daily living (APPADL)

The ability to perform physical activities of daily living (APPADL) contains seven items that assess how difficult it is for patients to engage in certain activities considered integral to normal daily life, such as walking, standing, and climbing stairs [10]. Items are scored on a five-point numeric rating scale, where 5 = “not at all difficult” and 1 = “unable to do.” A raw overall score is calculated by summing scores of the seven items and applying a linear transformation to yield a raw score ranging from 0 to 100. Higher scores indicate better ability to perform activities of daily living, and the items apply to the present.

#### Impact of weight on quality of life-lite clinical trials version (IWQOL-Lite-CT)

The impact of weight on quality of life-lite clinical trials version (IWQOL-Lite-CT) is a 20-item, obesity-specific PROM developed for use in obesity clinical trials. It assesses three primary composites of obesity-related health-related quality of life (HRQoL): physical (seven items), psychosocial (13 items), and a five-item subset of the physical composite—the physical function composite score. Items in the physical function composite describe physical impacts related to general and specific physical activities. All items are rated on either a five-point frequency scale (“never” to “always”) or a five-point truth scale (“not at all true” to “completely true”) [11, 12].

The IWQOL-Lite-CT composites and total scores are scored by averaging the responses of items within each scale, and then multiplying by the total number of items in the scale. Higher scores indicate better quality of life, and the recall period is 1 week.

### Analyses

Anchor-based methods, recommended by the United States (US) Food and Drug Administration (FDA) to interpret PROM scores, were considered the primary approach, and distribution-based methods, recommended as supportive, were secondary [13, 14]. Anchor-based methods are those that explore the association between the targeted concept of a PROM (in this case, self-perception associated with one’s body weight, as measured by the IW-SP) and the same or a closely related concept measured by an independent instrument, the anchor. Anchors are typically single-item stems with a response scale that has descriptions accompanying each response option, so that meaningfulness to patients can be interpreted. Using this method, changes seen in the PROM are compared—or *anchored*—to changes on the anchoring instrument. An anchor must be conceptually or logically related to the concept measured by the PROM of interest and show an association with that PROM.

Analyses estimating an MIC for the IW-SP used SURPASS-2 clinical trial data [9]. The SURPASS-2 study was an open-label, 40-week phase 3 trial of patients with T2D randomly assigned to receive tirzepatide at a dose of 5 mg, 10 mg, or 15 mg or semaglutide at a dose of 1 mg. The SURPASS-2 trial did not include a global scale related to patient impressions of change or severity in self-perception of body weight. In the absence of a specific anchor, the current study used multiple exploratory anchors to gather a body of evidence supporting a meaningful change in the IW-SP. The exploratory anchors that were considered included weight loss and scales from conceptually related PROMs that were completed by participants in the trial, the APPADL and the three composites and total score of the IWQOL-Lite-CT. In measuring one’s perceived quality of life (QoL) associated with body weight, the IWQOL-Lite-CT was seen as logically related to self-perception of body weight. The APPADL, in measuring ADLs associated with obesity, is likely more distally related to self-perception and body weight, but a relationship may exist such that those losing weight improve in their ADLs as well as in their self-perception of body weight. Both PROMs have established MICs in people with either T2D or obesity. In addition, the IWQOL-Lite-CT also has two individual items that measure self-perceptions associated with body weight that were used as anchors: (Item 7) *I feel less confident because of my weight* (never, rarely, sometimes, usually, always) and (Item 20) *I feel frustrated or upset with myself about my weight* (not at all true, a little true, moderately true, mostly true, completely true).

Both anchor- and distribution-based approaches were used for the current analyses, first in 2/3 of the sample (estimation sample), then these initial results were confirmed in the other 1/3 of the sample (confirmation sample). The estimation and confirmation subsamples were created by random assignment. As most participants in the SURPASS-2 trial lost weight (approximately 73%), these analyses focused on change in terms of weight loss and improvement in PROM scores. Note that the process of deriving anchors was only performed during the estimation stage.

All analyses were conducted without consideration for treatment assignment in the trial, as the objective was to assess the meaningfulness of improvement in perception of body weight, irrespective of what drives weight loss. Change for all analyses was calculated using data from baseline and the trial endpoint (week 40), as these were the only visits where PROs were administered. All analyses were conducted using SAS Software (version 9.4).

#### Identifying potential anchors

The first step was defining prospective anchors, which could be used to group trial participants in terms of meaningful improvement. MIC values for weight loss from the literature show 5% and 10% change as frequently used indices of meaningful change in body weight [10, 15, 16], so both estimates were considered. The MIC for the APPADL was previously estimated as a range based on multiple approaches, from 6 to 14 points on a scale from 0 to 100 [10]. The midpoint of this range, 10 points, was used in the current analysis. The MICs for the IWQOL-Lite-CT were established by the developers as follows: physical composite (13.5 points), physical function composite (14.6), psychosocial composite (16.2 points), and total score (16.6 points); all scales range from 0 to 100 [17]. Change in Item 7 and Item 20 of the IWQOL-Lite-CT are discrete integers ranging from − 4 to 4, so the smallest change possible (one point) was used.

Finally, the percent of weight change that corresponds to the MICs of the APPADL and the IWQOL-Lite-CT Scales were estimated. These values were compared with the 5% and 10% values. These were estimated using regression, where change in the respective scale was the independent variable, percent change in body weight was the dependent variable, and the value of percent change in body weight predicted by each respective scale’s MIC was the value considered as a potential anchor.

#### Selection of anchors

First, only variables showing a minimum relationship of |0.30| (Spearman’s rho) in change [18] distributions (baseline to trial endpoint) were further included as anchors in the subsequent analyses. Second, redundant anchors for weight were eliminated. That is, potential anchors that grouped subjects by weight loss were eliminated if they converged on the same quantity of weight loss For example, seven anchors using percent change in weight—two from the literature [10, 15, 16] and five predicted by scale MICs—were explored as potential anchors for further analyses. A ≤ 5% point was identified a priori as the cut-off for redundant anchors, as 5% was the smallest MIC for weight change identified in our literature searches.

#### Application of anchors to form groups

The next step was to apply the anchors to form participant groups based on change in MIC. As the objective was to estimate the *minimum* meaningful change in the IW-SP, these analyses were performed using *half* MICs for all anchors, save for the IWQOL-Lite-CT items. Half MIC intervals were used in order to define a reasonable range within each change category for each of the anchor variables, as few (if any) people would have experienced exactly a 1 MIC change for each anchor variable. The intervals around the half MICs were shifted, such that groupings encompassed 1.0 times each anchors’ actual MIC (Table 1—middle column).

**Table 1:** Interval groupings of participants for IW-SP MIC estimation analyses

Group	Continuous anchors with existing MICs^a^	Individual items^b^
Group 1	< 0.25 MIC (no change)	0 category change (no change)
Group 2	0.25 MIC to < 0.75 MIC improvement	+ 1 category improvement*
Group 3	0.75 MIC to < 1.25 MIC improvement*	+ 2 category improvement
Group 4	1.25 MIC to < 1.75 MIC improvement	+ 3 category improvement
Group 5	1.75 MIC to < 2.25 MIC improvement	+ 4 category improvement
Group 6	2.25 MIC to < 2.75 MIC improvement	
Group 7	> 2.75 MIC improvement	

*IW-SP* Impact of Weight on Self-perception Questionnaire, *MIC* minimal important change

*The estimated change in IW-SP that corresponded to this level of change for each anchor variable were of primary interest in the current study as this represents the change in the IW-SP score that corresponds to a 1 MIC improvement in the continuous anchors, or a 1-point improvement in the individual items

^a^Each group corresponds to change in units of respective PROM MIC as follows: APPADL MIC = 10 points; IWQOL-Lite-CT physical composite MIC = 13.5 points, physical function MIC = 14.6, psychosocial composite MIC = 16.2 points, total score MIC = 16.6 points

^b^Groups correspond to all possible improvements in item response scale

For the IWQOL-Lite-CT individual items, the “no change” group comprised participants indicating 0 points of change between baseline and trial endpoint, and the additional groups indicated improvements of 1–4 points between baseline and trial endpoint (five groups in all; Table 1—last column).

#### Assessing meaningful change and responsiveness

Once anchors were selected, group differences by anchor category were tested to establish responsiveness with the prediction that group differences on the IW-SP scores would be a function of differences in the conceptually related anchors. Responsiveness refers to the extent to which the instrument can detect true change in participants known to have changed on the concept of interest [19]. IW-SP responsiveness analyses compared change scores between participants with different degrees of anchor change using general linear models (analysis of covariance [ANCOVA]), controlling for age, gender, and baseline body mass index for each anchor.

These analyses also allowed assessment of the IW-SP’s ability to reliably discriminate groups defined by differing increments of change, adding evidence for the reliability of the estimated MIC.

#### Distribution-based approaches

Distribution-based methods for estimating the magnitude of meaningful change in PROM scores utilize statistical parameters from the clinical trial population, where a PROM change score is expressed relative to some measure of variability.

The distribution-based approach was a calculation of standard deviation (SD) units using the baseline score. The half SD has been shown to provide a reasonable approximation of a meaningful change in PROMs [20]. The standard error of measurement (SEM) [21] was not included, as the time interval between baseline and trial endpoint was too long to estimate test–retest reliability, which is required for calculation of the SEM.

#### Triangulation

A single estimate for an MIC for improvement in the IW-SP was achieved by triangulating findings of the anchor- and distribution-based analyses, supported by visualization of data on cumulative distribution function and probability density function plots [18, 22, 23]. Special attention was paid to the IW-SP change score that corresponded to a one-category improvement (*0.75 to* < *1.25 MIC* improvement) when there was a statistically significant group difference between IW-SP scores corresponding to that anchor category and the adjacent “no change” anchor category. MIC estimates for the IW-SP from the confirmation and estimation stages are presented and discussed together, allowing the extent of the convergence of estimates to be evaluated. That is, comparison of the estimation and confirmation stage estimates is itself a form of triangulation.

The MIC for improvement in the IW-SP total score at this stage was expressed in terms of the transformed scale and the raw scale. The latter is more interpretable as it aligns directly with the actual IW-SP responses. Following this process, a final MIC estimate was produced and rounded to the nearest mathematically possible change value for the IW-SP total score, both in terms of raw and transformed scales.

## Results

### Sample description

The total sample was *N* = 1878: *n* = 1252 in the estimation group and *n* = 626 in the confirmation group (Table 2). The groups were similar in terms of sociodemographics, IW-SP, and anchor scores (Tables 2, 3, S1).

**Table 2:** Sociodemographic and clinical characteristics for the total sample, MIC estimation group, and MIC confirmation group

	Total (*N* = 1878)	MIC estimation (*N* = 1252)	MIC confirmation (*N* = 626)
Sex, *n* (%)			
Male	882 (47.0%)	591 (47.2%)	291 (46.5%)
Female	996 (53.0%)	661 (52.8%)	335 (53.5%)
Ethnicity, * n* (%)			
Hispanic or Latino	1317 (70.1%)	869 (69.4%)	448 (71.6%)
Not Hispanic or Latino	561 (29.9%)	383 (30.6%)	178 (28.4%)
Race, * n* (%)			
American Indian or Alaska Native	208 (11.1%)	129 (10.3%)	79 (12.6%)
Asian	25 (1.3%)	16 (1.3%)	9 (1.4%)
Black or African American	79 (4.2%)	61 (4.9%)	18 (2.9%)
Native Hawaiian or other Pacific Islander	3 (0.2%)	1 (0.1%)	2 (0.3%)
White	1551 (82.6%)	1036 (82.7%)	515 (82.3%)
Multiple	12 (0.6%)	9 (0.7%)	3 (0.5%)
Age (years)			
* n*	1878	1252	626
Mean	56.6	56.5	56.7
SD	10.4	10.5	10.3
Minimum	21	21	24
Median	57	57	57
Maximum	91	82	91
Weight			
* n*	1878	1252	626
Mean	93.7	93.7	93.8
SD	21.9	22.0	21.6
Minimum	50	53	50
Median	90	90	90
Maximum	222	222	198
Baseline BMI			
* N*	1878	1252	626
Mean	34.2	34.1	34.5
SD	6.9	6.8	7.1
Minimum	23	24	23
Median	33	33	33
Maximum	89	89	86
Baseline A1C			
* N*	1878	1252	626
Mean	8.3	8.3	8.3
SD	1.0	1.0	1.0
Minimum	6	6	6
Median	8	8	8
Maximum	12	12	12

*A1C* glycated hemoglobin, *BMI* body mass index, *MICMIC* minimal important change, *SD* standard deviation

**Table 3:** Descriptive summary of PROM and weight change^a^ scores

Score change	Stage	*N*	Mean change (SD)	Range (minimum–maximum)	Missing (%)	*N*, *ρ* ^b^
IW-SP Score	Estimation	1200	10.0 (26.3)	− 200	52 (4.2%)	–
	Confirmation	603	11.7 (27.2)	− 200	23 (3.7%)	
IWQOL-Lite-CT Physical composite	Estimation	1197	8.7 (18.9)	− 150	55 (4.4%)	1799, 0.40****
	Confirmation	602	9.7 (20.2)	− 150	24 (3.8%)	
IWQOL-Lite-CT Physical Function composite	Estimation	1197	9.5 (20.5)	− 150	55 (4.4%)	1799, 0.40****
	Confirmation	602	10.6 (22.1)	− 160	24 (3.8%)	
IWQOL-Lite-CT Psychosocial composite	Estimation	1197	8.2 (18.3)	− 167.3	55 (4.4%)	1799, 0.57****
	Confirmation	602	9.8 (20.2)	− 134.6	24 (3.8%)	
IWQOL-Lite-CT Total Score	Estimation	1196	8.4 (16.9)	− 155.1	56 (4.5%)	1798, 0.56****
	Confirmation	602	9.8 (18.5)	− 127.5	24 (3.8%)	
APPADL Score	Estimation	1199	5.8 (17.7)	− 128.5	53 (4.2%)	1801, 0.35****
	Confirmation	602	6.3 (19.4)	− 150	24 (3.8%)	
IWQOL-Lite-CT Item 7	Estimation	1197	− 0.3 (1.1)	− 8	55 (4.4%)	1799, − 0.50****
	Confirmation	602	− 0.4 (1.1)	− 8	24 (3.8%)	
IWQOL-Lite-CT Item 20	Estimation	1195	− 0.3 (1.2)	− 8	57 (4.6%)	1797, − 0.49****
	Confirmation	602	− 0.5 (1.2)	− 7	24 (3.8%)	
Weight (KG, % Change)	Estimation	1188	− 9.0 (7.4)	− 53.7	64 (5.1%)	1784, − 0.23****
	Confirmation	598	− 9.9 (7.3)	− 42.1	28 (4.5%)	

*APPADL* ability to perform physical activities of daily living, *IW-SP* Impact of Weight on Self-perception, *IWQOL-Lite-CT* Impact of weight on quality of life-lite clinical trials version, *SD* standard deviation

*p < 0.05; **p < 0.01; ***p < 0.001; ****p < 0.0001

^a^Baseline to endpoint (week 40)

^b^Spearman’s rho, change in prospective anchors correlated with change in IW-SP scores, baseline to endpoint

As more than 25% of participants had baseline scores at ceiling for both the estimation and confirmation stages (31.7% and 31.2%, respectively), a sensitivity analysis was performed wherein meaningful change and responsiveness analyses were repeated after removing all subjects with baseline IW-SP scores at ceiling. Analyses were performed using the entire sample; results can be found in Supplement 1.

### Responsiveness and meaningful change analyses

#### Anchor selection and anchor-based analyses

Regression analyses to identify change in body weight corresponding to each of the anchor scale’s MICs converged on a similar estimate, falling between 10.2 and 10.8%, so only 10% change was included in the subsequent analyses. The correlations between change distributions for each potential anchor and the IW-SP showed that all prospective anchors exceeded the minimum requirement of *ρ* =|0.30| except for percent change in weight, which did not reach the threshold (*ρ* = − 0.23; Table 3). The relationships mostly varied as a function of conceptual relatedness, as the APPADL was the weakest after percent change in weight (*ρ* = 0.35), and the IWQOL-Lite-CT Psychosocial composite was the strongest (*ρ* = 0.57). While percent change in weight did not meet criteria, and is not considered an anchor, it was included in subsequent reporting as a reference to the prior literature.

Following these analyses, the exploratory anchors submitted to meaningful change, responsiveness, and triangulation analyses were as follows: IWQOL-Lite-CT Physical composite, Physical Function composite, Psychosocial composite, and Total Score, and the IWQOL-Lite-CT Item 7 and Item 20, and the APPADL.

With few exceptions, the IW-SP total score changed in the expected direction and consistently across anchor categories for both the estimation (Table 4) and confirmation (Table S2) analyses, where self-perception regarding weight improved as anchor categories improved. All prospective anchoring scales showed significant omnibus effects across anchor categories (all *p* < 0.0001) for both the estimation (Table 4) and confirmation (Table S2) groups. The important anchor category corresponding to 1 MIC of change (*0.75 to* < *1.25 MIC*) showed relatively high variability at confirmation and estimation stages, as CVs were near 1 or > 1 for the anchor categories based on summary scores (0.89 to 1.32), though CVs for individual Item 7 and Item 20 were lower (0.59 to 0.66).

**Table 4:** Responsiveness^a^ of IW-SP by half MIC anchor groupings, all anchors, estimation group

Anchor	Mean type	< 0.25 MIC		0.25 to < .0.75 MIC		0.75 to < 1.25 MIC		1.25 to < 1.75 MIC		1.75 to < 2.25 MIC		2.25 to < 2.75 MIC		> 2.75 MIC		Overall F-test ^b^		Pairwise Comparison ^c^
		*N*	Mean	*N*	Mean	*N*	Mean	*N*	Mean	*N*	Mean	*N*	Mean	*N*	Mean	F	*p*-value	(*p*-value )
IWQOL-Lite-CT Physical composite MIC	Raw Mean (SD)	113	5.7 (9.9)	186	12.0 (17.1)	152	16.8 (19.8)	105	21.9 (21.7)	75	26.2 (19.6)	52	32.9 (23.3)	90	37.4 (25.9)			2**,3***,4***,5***,6***,8*,9*** ,10***,11***,14**,15***,18***
	LS Mean (SE)	113	6.9 (1.8)	186	12.2 (1.4)	152	16.9 (1.5)	105	21.0 (1.9)	75	25.7 (2.2)	52	31.1 (2.7)	90	35.6 (2.0)	27.49	< .0001	
IWQOL-Lite-CT Physical Function composite MIC	Raw Mean (SD)	162	6.4 (11.0)	214	12.7 (16.4)	98	19.6 (20.9)	121	21.5 (21.2)	50	27.0 (21.9)	71	30.4 (23.8)	76	39.7 (25.3)			2**,3***,4***,5***,6***,8*,9**,10 ***,11***,15***,18***
	LS Mean (SE)	162	7.8 (1.5)	214	12.6 (1.3)	98	19.0 (1.9)	121	21.0 (1.7)	50	26.0 (2.6)	71	29.3 (2.2)	76	37.6 (2.2)	29.16	< .0001	
IWQOL-Lite-CT Psychosocial composite MIC	Raw Mean (SD)	213	7.9 (14.8)	172	12.6 (15.1)	122	20.8 (18.5)	89	29.0 (18.2)	57	33.5 (20.6)	40	46.7 (19.6)	49	54.9 (21.4)			2***,3***,4***,5***,6***,7*,8*** ,9***,10***,11***,13**,14***,15 ***,17***,18***,19*,20***
	LS Mean (SE)	213	8.3 (1.2)	172	12.8 (1.3)	122	20.4 (1.6)	89	28.2 (1.8)	57	32.7 (2.3)	40	45.4 (2.7)	49	53.8 (2.5)	56.53	< .0001	
IWQOL-Lite-CT Total Score MIC	Raw Mean (SD)	181	5.8 (10.5)	192	13.8 (16.5)	162	20.4 (19.3)	83	28.6 (18.4)	66	37.5 (20.1)	27	40.7 (13.5)	39	60.3 (22.6)			1**,2***,3***,4***,5***,6***,8*** ,9***,10***,11***,13***,14*** ,15***,18***,20***,21**
	LS Mean (SE)	181	6.2 (1.3)	192	14.0 (1.2)	162	20.0 (1.3)	83	27.9 (1.8)	66	36.6 (2.1)	27	39.3 (3.2)	39	58.7 (2.7)	59.25	< .0001	
APPADL MIC	Raw Mean (SD)	127	9.5 (16.3)	180	15.8 (21.1)	61	18.3 (19.7)	59	17.8 (22.0)	96	21.4 (19.1)	48	20.8 (18.8)	128	35.5 (25.1)			4*,6***,11***,15**,18***,20** ,21**
	LS Mean (SE)	127	9.9 (1.8)	180	16.5 (1.5)	61	18.6 (2.6)	59	17.2 (2.6)	96	21.0 (2.0)	48	18.9 (2.9)	128	33.2 (1.8)	18.62	< .0001	
Weight change MIC (10%)	Raw Mean (SD)	99	12.4 (17.1)	252	13.3 (17.0)	250	15.7 (20.8)	181	21.9 (24.1)	63	31.1 (25.4)	35	26.9 (22.1)	18	41.2 (24.2)			4***,6**,8*,9***,11**,13***,15**
	LS Mean (SE)	99	12.7 (2.0)	252	13.7 (1.3)	250	15.6 (1.3)	181	21.5 (1.5)	63	29.2 (2.5)	35	24.2 (3.4)	18	37.0 (4.7)	17.81	< .0001	

Anchor	Mean Type	0 Point		1 point		2 points		3 points		4 points						*F*	*p*-value	(*p*-value)
		*N*	Mean	*N*	Mean	*N*	Mean	*N*	Mean	*N*	Mean							
IWQOL-Lite-CT Item 7 ^d,e^	Raw Mean (SD)	541	10.0 (15.9)	182	22.6 (18.2)	123	34.5 (23.0)	31	48.4 (24.6)	17	47.1 (34.1)							1***,2***,3***,4***,5***,6***,7** *,8**
	LS Mean (SE)	541	10.7 (0.8)	182	21.3 (1.3)	123	33.0 (1.6)	31	46.4 (3.2)	17	44.4 (4.4)					56.83	< .0001	
IWQOL-Lite-CT Item 20 ^d,e^	Raw Mean (SD)	557	9.9 (15.9)	167	24.2 (18.8)	92	33.3 (20.0)	45	45.6 (20.5)	26	51.0 (30.5)							1***,2***,3***,4***,5**,6***,7*** ,8*,9***
	LS Mean (SE)	557	10.4 (0.7)	167	23.4 (1.4)	92	31.9 (1.8)	45	43.1 (2.7)	26	49.4 (3.5)					63.13	< .0001	

*APPADL* ability to perform physical activities of daily living, *IWQOL-Lite-CT* Impact of weight on quality of life-lite clinical trials version, *LS* least squares, *MIC* minimal important change, *SD* standard deviation, *SE* standard error

^a^Mean change, Baseline to endpoint (week 40)

^b^ANCOVA model with covariates of age, gender, and baseline body mass index

^c^Pairwise comparisons between means were performed using Scheffe's test adjusting for multiple comparisons: 1: < 0.25 MIC vs. 0.25 to < .75 MIC; 2: < 0.25 MIC vs. 0.75 to < 1.25 MIC; 3: < 0.25 MIC vs. 1.25 to < 1.75 MIC; 4: < 0.25 MIC vs. 1.75 to < 2.25 MIC; 5: < 0.25 MIC vs. 2.25 to < 2.75 MIC; 6: < 0.25 MIC vs. > 2.75 MIC; 7: 0.25 to < .75 MIC vs. 0.75 to < 1.25 MIC; 8: 0.25 to < .75 MIC vs. 1.25 to < 1.75 MIC; 9: 0.25 to < .75 MIC vs. 1.75 to < 2.25 MIC; 10: 0.25 to < .75 MIC vs. 2.25 to < 2.75 MIC; 11: 0.25 to < .75 MIC vs. > 2.75 MIC; 12: 0.75 to < 1.25 MIC vs. 1.25 to < 1.75 MIC; 13: 0.75 to < 1.25 MIC vs. 1.75 to < 2.25 MIC; 14: 0.75 to < 1.25 MIC vs. 2.25 to < 2.75 MIC; 15: 0.75 to < 1.25 MIC vs. > 2.75 MIC; 16: 1.25 to < 1.75 MIC vs. 1.75 to < 2.25 MIC; 17: 1.25 to < 1.75 MIC vs. 2.25 to < 2.75 MIC; 18: 1.25 to < 1.75 MIC vs. > 2.75 MIC; 19: 1.75 to < 2.25 MIC vs. 2.25 to < 2.75 MIC; 20: 1.75 to < 2.25 MIC vs. > 2.75 MIC; 21: 2.25 to < 2.75 MIC vs. > 2.75 MIC. *p < 0.05; **p < 0.01; ***p < 0.001

^d^IWQOL-Lite-CT individual items will include five groups corresponding to 0 points (no change) and 1, 2, 3, and 4 points of improvement

^e^Pairs for IWQOL-Lite-CT individual items: 1: No change vs. 1 point improvement, 2: No change vs. 2 points improvement, 3: No change vs. 3 points improvement, 4: No change vs. 4 points improvement, 5: 1 point improvement vs. 2 points improvement, 6: 1 point improvement vs. 3 points improvement, 7: 1 point improvement vs. 4 points improvement, 8: 2 points improvement vs. 3 point improvement, 9: 2 points improvement vs. 4 points improvement, 10: 3 points improvement vs. 4 points improvement

Pairwise follow-up tests for significant omnibus effects, however, differentiated the prospective anchor scales. The APPADL MIC and weight change (5% and 10%) only showed significant pairwise differences when comparing patients categorized as not improving or improving very little vs. those who improved markedly (e.g., improvement by < *0.25 MIC* anchor category vs. improvement by > *2.75 MIC* anchor category).

The composites and items of the IWQOL-Lite-CT, by contrast, revealed better responsiveness of the IW-SP, as pairwise comparisons using anchors based on these scales were able to significantly differentiate IW-SP change scores when comparing most anchor-based groupings of participants. This includes the comparisons of the IW-SP scores corresponding to < *0.25 MIC* vs. *0.75 to* < *1.25 MIC* (i.e., Group 1 vs. Group 3, Table 1) for all of the IWQOL-Lite-CT composites in the estimation analysis, and for the IWQOL-Lite-CT Psychosocial composite and IWQOL-Lite-CT total score for the confirmation analysis.

Of the IWQOL-Lite-CT composites, however, only the participant groupings based on the IWQOL-Lite-CT Psychosocial composite—Item 7 (*I feel less confident because of my weight*) and Item 20 (*I feel frustrated or upset with myself about my weight*)—were able to differentiate IW-SP change scores from adjacent anchor categories. IW-SP scores corresponding to the anchor category of *0.75 to* < *1.25 MIC* of the psychosocial score were significantly improved compared with those corresponding to the < *0.25 to* < *0.75 MIC* category (i.e., Group 2 vs. Group 3, Table 1), and this was the case for both the estimation and confirmation analyses (all *p* < 0.05).

Similarly, IW-SP change scores corresponding to a 1-point improvement on both Item 7 and Item 20 were significantly improved compared to scores corresponding to no change on the items, and this was the case for both the estimation and confirmation analyses (all *p* < 0.01). That is, the scores corresponding to the category reflecting the minimal amount of improvement was significantly improved compared to the scores corresponding to the category reflecting no change. Most other comparisons for Item 7 and Item 20 showed significant pairwise comparisons for both the estimation and confirmation groups. These results support that these two items are likely the best anchors available. This is not surprising given that these two items are also most closely conceptually aligned with the IW-SP.

While all anchors showed evidence for the responsiveness of the IW-SP to change, these results indicate that the comparisons of anchor categories representing minimal possible change were reliable for conceptually related scales: the IWQOL-Lite-CT Psychosocial composite, and the two items comprising that scale, Item 7 and Item 20.

### Distribution-based approaches

Distribution-based estimates can be found in Table 5. Estimates are very similar across the estimation and confirmation groups. These results are supportive of those found in anchor-based analyses, where half SD is slightly lower at approximately 15 points.

**Table 5:** Triangulation: IW-SP Change Scores Corresponding to Minimal Important Change^a^ in Anchor- and Distribution-Based Analyses

Scale	Transformed score improvement (0–100 points)			IW-SP raw scale units improvement (1–5-point scale)			Total # of response categories Improved across all three items		
	Estimation	Confirmation	Difference	Estimation	Confirmation	Difference	Estimation	Confirmation	Difference
IWQOL-Lite-CT Physical ^b^	16.80	16.70	0.10	0.67	0.67	0.00	2.03	2.03	0.00
IWQOL-Lite-CT Physical^b^ Function	19.60	21.10	− 1.50	0.78	0.84	− 0.06	2.36	2.55	− 0.19
IWQOL-Lite-CT Psychosocial^b,c^	20.80	22.30	− 1.50	0.83	0.89	− 0.06	2.52	2.70	− 0.18
IWQOL-Lite-CT Total^b^	20.40	20.80	− 0.40	0.82	0.83	− 0.01	2.48	2.52	− 0.04
IWQOL-Lite-CT Item #7^b,c^	22.60	25.90	− 3.30	0.90	1.04	− 0.14	2.73	3.15	− 0.42
IWQOL-Lite-CT Item #20^b,c^	24.20	26.90	− 2.70	0.97	1.08	− 0.11	2.94	3.27	− 0.33
APPADL Total	18.30	17.30	1.00	0.73	0.69	0.04	2.21	2.09	0.12
Distribution, 1/2 SD	15.03	15.30	− 0.27	0.60	0.61	− 0.01	1.82	1.85	− 0.03
*Weight change (10%)*^d^	*15.70*	*19.10*	− *3.40*	*0.63*	*0.76*	− *0.13*	*1.91*	*2.30*	− *0.39*
Mean (SD)	19.72 (3.0)	20.79 (4.2)	− 1.07 (1.5)	0.79 (0.1)	0.83 (0.2)	− 0.04 (0.1)	2.39 (0.4)	2.52 (0.5)	− 0.13 (0.2)
Minimum score	15.03	15.30	− 3.30	0.60	0.61	− 0.14	1.82	1.85	− 0.42
Maximum score	24.20	26.90	1.00	0.97	1.08	0.04	2.94	3.27	0.12

*APPADL* ability to perform physical activities of daily living, *IW-SP* Impact of Weight on Self-perception Questionnaire, *IWQOL-Lite-CT* Impact of weight on quality of life-lite clinical trials version, *SD* standard deviation

^a^Corresponding to *0.75 to* < *1.25 MIC* for scales, 1-point improvement for IWQOL Items 7 and 20, and half SD for distribution-based analysis

^b^Showed significant difference in responsiveness analysis between *0.75 to* < *1.25 MIC* vs. < *0.25 MIC*

^c^Showed significant difference in responsiveness analysis between adjacent anchor categories: *0.75 to* < *1.25 MIC* vs. *0.25 to* < *.75 MIC* for the IWQOL-Lite-CT Psychosocial Scale; No change vs. 1-point improvement for the IWQOL-Lite-CT Items

^d^Weight Change included for reference, but not included in calculations as it did not meet a priori criteria for inclusion as anchor

### Triangulation

Triangulation results can be found in Table 5, which shows the IW-SP change score that corresponds to the *0.75 to* < *1.25 MIC* of change in each respective anchor, 1-point improvement compared to no change for the IWQOL-Lite-CT items, and the distribution-based estimate of half SD. That is, this table shows the change in the IW-SP total score that corresponds to the minimum change for each anchor.

Results in Table 5 are given in 0–100 transformed score units as well as in IW-SP Total raw scale units (i.e., the mean of the three IW-SP items, each of which are on a 1–5-point scale). Triangulation is discussed in terms of raw units of the IW-SP. While the transformed score is useful for easy calculation and formation of heuristics, it is more intuitive to present evidence for the MIC in terms of the actual participant responses. Change in the IW-SP is also included in terms of the total number of changes in response categories across the three IW-SP items (raw score/0.33) needed to produce the corresponding total score change.

There is a high degree of consistency between the estimation and confirmation groups, with no estimate differing by more than 0.2 points, and the average difference is near 0. Estimates of the MIC for improvement in the IW-SP total score range from 0.60 points to 1.08 points across 16 estimates and two approaches (anchor-based and distribution-based).

When examining the responsiveness results, it’s clear that the IW-SP was more sensitive to small anchor category changes when anchored by the composites of IWQOL-Lite-CT, and sensitivity was greatest when anchored by the Psychosocial composite and two of the most relevant items that comprise that composite (Item 7 and Item 20). This is as expected as these are conceptually the closest to the IW-SP. However, the range of MIC estimates when considering these scales does not markedly differ from the other estimates, ranging from 0.83 points to 1.08 points for anchor-based estimates.

**Fig. 1:**
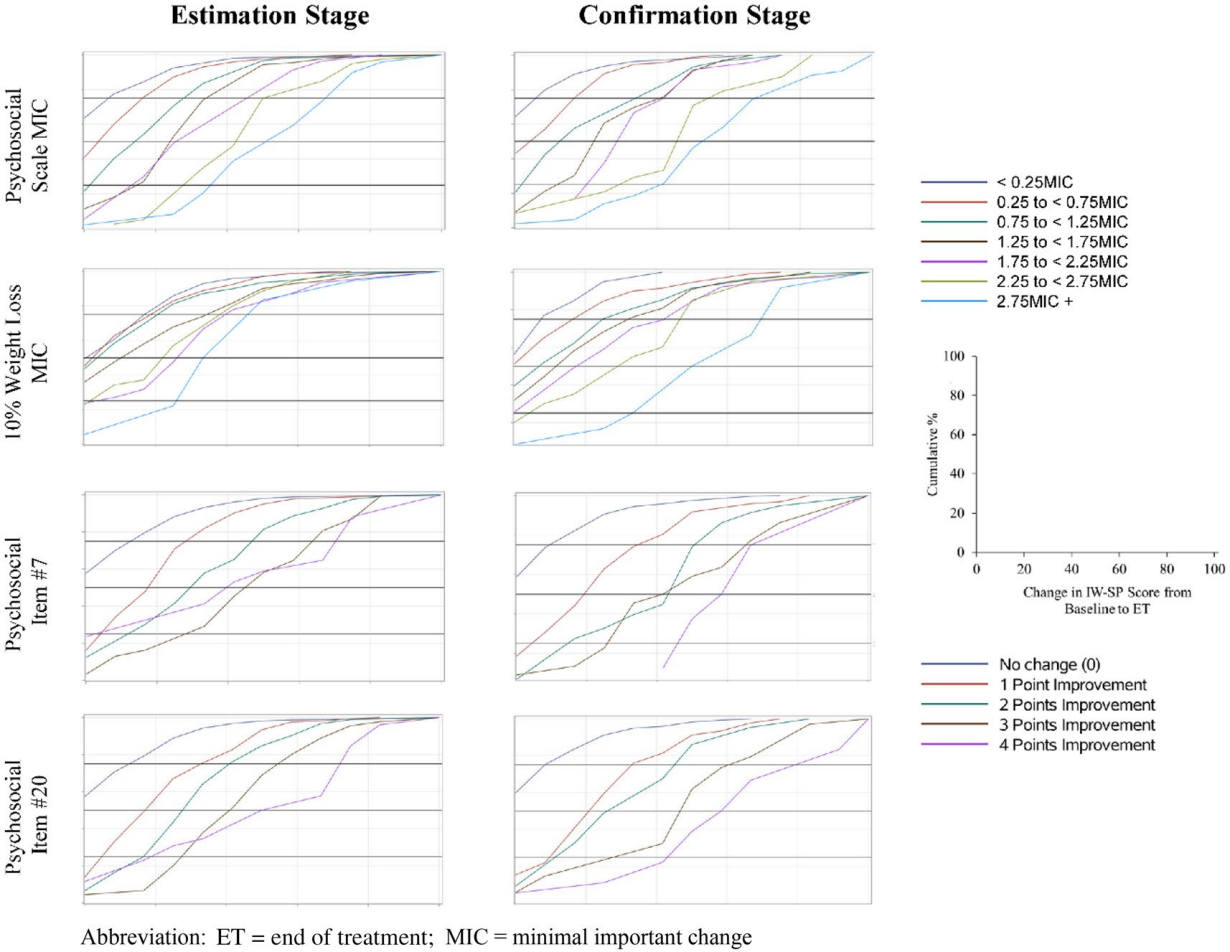
CDF plots: change in IW-SP score by select exploratory anchors. *MIC* minimal important change

Rounding up to be conservative, an MIC of a *1-point* improvement for the IW-SP total raw score is supported by these analyses, equivalent to a *25-point*^1^ improvement of the transformed score. This is the equivalent of 3 points of response category improvement across the three items of the three IW-SP items (i.e., this corresponds to patients improving on average by 1 category across each of the three IW-SP questions). This estimate is also supported by CDF plots; Fig. 1 shows the CDF plots for select anchors, including the Psychosocial Scale and Items 7 and 20 from the IWQOL-Lite-CT (%weight loss is included as a reference). The degree to which these curves are separate from one another, and do not overlap, reflects how distinct the change scores from one anchor category are from the others.

## Discussion

Both the estimation and confirmation stages of the current study arrived at an MIC of a 25-point improvement for the IW-SP, corresponding to a one-category improvement, on average, across each of the three IW-SP questions. While these estimates were stable across the study stages, both groups showed considerable individual variability where the impact of body weight on body image is concerned. Considering the convergence of estimates for the most conceptually relevant anchors, the IWQOL-Lite-CT Psychosocial Scale and Items 7 and 20, the MIC might also be expressed as a range from 0.83 to 1.08 in raw scores, or 21 to 27 transformed points, as the more single point estimate above may miss meaningful change that is present.

Many participants did not report having any issues with their self-perception, even when they had very high body mass indexes. It is important to note that for these people, or any people with scores greater than 75 on the IW-SP at baseline, there is insufficient room to demonstrate a meaningful change on the IW-SP. Accordingly, future studies using the IW-SP as a key endpoint will need to consider baseline IW-SP score as an inclusion criteria to ensure that participants have scores that will allow for a meaningful change in the IW-SP (i.e., they must have scores < 75 at baseline).

The results of this study should be interpreted with consideration for the following limitations. First, there was not a conceptually *a-priori* appropriate global scales to anchor the IW-SP. In addition, it was an exploratory post hoc analysis of available clinical trial data, though the process for identifying and selecting anchors was prespecified. Use of clinical trial data for estimating psychometric properties is a common approach in PROM development and validation. This study focused on the MIC for improvement, as the MIC for deterioration in self-perception, measured by the IW-SP due to weight gain, cannot be established based on the results of the current study. Finally, it is important to note that this MIC is intended for classifying minimal within-person change over time, for example as a threshold for determining the number of responders, rather than as a value for interpreting between-group mean differences [8].

Interestingly, the conceptually related IWQOL-Lite-CT Items 7 and 20 showed greater sensitivity in responsiveness analyses but also showed higher MIC estimates. This may not be surprising given that the PROM-derived anchors used existing MIC estimates reflect the smallest meaningful difference to patients, and are comprised multiple items measuring a range of the facets associated with the respective concept. The single items on the other hand used the smallest change possible on their response scale, which may not represent a minimum change to patients, and were limited to the concepts of *confidence* (Item 7) and *frustration* (Item 20) with body weight. It may be that the response scales are not sensitive enough or do not capture the full range of concepts related to self-image associated with body weight.

The results of this study yield an estimate of the MIC for the IW-SP, which was derived based on a triangulation of many estimates from multiple anchors and distribution-based estimates. These estimates all converged quite closely and consistently on the identified value and reflect reasonable changes in the IW-SP scale (about a one-category change across each of the three items) that could conceivably reflect meaningful improvements from the patient perspective.

### Supplementary Information

Below is the link to the electronic supplementary material.

Supplementary file1 (DOCX 77 kb)

## Footnotes

1 Transformed score = ([observed raw score – lowest possible raw score])/possible raw score range)  × 100.
