# Supplementary material for: Estimating meaningful change for The Impact of Weight on Self-Perception (IW-SP) questionnaire among people with type 2 diabetes
Source: Qual Life Res. 2023 Jul 25;32(12):3359–71. doi: 10.1007/s11136-023-03476-0 (PMC10624730; doi:10.1007/s11136-023-03476-0)
Supplement: Supplementary file 1 — Supplementary file1 (DOCX 77 kb) [file 11136_2023_3476_MOESM1_ESM.docx]

#

# Supplementary Material

**Supplementary Table S1. Descriptive Summary of PROM Scores and Weight**

| **Score** | **Timepoint** | **Stage** | **N** | **Mean (SD)** | **Range (Minimum-Maximum)** | **Floor (%)** | **Ceiling (%)** |
| --- | --- | --- | --- | --- | --- | --- | --- |
| IW-SP Score | Baseline | Estimation | 1,252 | 69.2 (29.9) | 0.0–100.0 | 46 (3.7%) | 397 (31.7%) |
|  |  | Confirmation | 626 | 68.9 (30.7) | 0.0–100.0 | 27 (4.3%) | 195 (31.2%) |
| IWQOL-Lite-CT Physical composite |  | Estimation | 1,252 | 64.6 (24.0) | 0.0–100.0 | 7 (0.6%) | 54 (4.3%) |
|  |  | Confirmation | 626 | 65.0 (25.1) | 0.0–100.0 | 4 (0.6%) | 29 (4.6%) |
| IWQOL-Lite-CT Physical Function composite |  | Estimation | 1,252 | 64.4 (25.3) | 0.0–100.0 | 10 (0.8%) | 105 (8.4%) |
|  |  | Confirmation | 626 | 64.8 (26.2) | 0.0–100.0 | 11 (1.8%) | 46 (7.3%) |
| IWQOL-Lite-CT Psychosocial composite |  | Estimation | 1,252 | 72.1 (23.7) | 0.0–100.0 | 1 (0.1%) | 45 (3.6%) |
|  |  | Confirmation | 626 | 71.7 (25.0) | 0.0–100.0 | 2 (0.3%) | 31 (5.0%) |
| IWQOL-Lite-CT Total Score |  | Estimation | 1,252 | 69.4 (22.2) | 0.0–100.0 | 1 (0.1%) | 16 (1.3%) |
|  |  | Confirmation | 626 | 69.3 (23.4) | 0.0–100.0 | 1 (0.2%) | 13 (2.1%) |
| APPADL Score |  | Estimation | 1,251 | 69.0 (23.0) | 0.0–100.0 | 6 (0.5%) | 103 (8.2%) |
|  |  | Confirmation | 625 | 68.5 (23.7) | 3.6–100.0 | 0 (0.0%) | 52 (8.3%) |
| IWQOL-Lite-CT Item 7 |  | Estimation | 1,252 | 2.0 (1.2) | 1.0–5.0 | 654 (52.2%) | 72 (5.8%) |
|  |  | Confirmation | 626 | 2.0 (1.3) | 1.0–5.0 | 324 (51.8%) | 39 (6.2%) |
| IWQOL-Lite-CT Item 20 |  | Estimation | 1,251 | 2.0 (1.4) | 1.0–5.0 | 680 (54.4%) | 113 (9.0%) |
|  |  | Confirmation | 626 | 2.1 (1.4) | 1.0–5.0 | 335 (53.5%) | 71 (11.3%) |
| Weight (KG) |  | Estimation | 1,252 | 93.7 (22.0) | 52.5–222.1 | 0 (0.0%) | 0 (0.0%) |
|  |  | Confirmation | 625 | 93.8 (21.6) | 50.1–198.2 | 0 (0.0%) | 0 (0.0%) |
| IW-SP Score | Endpoint | Estimation | 1,200 | 78.9 (25.6) | 0.0 –100.0 | 20 (1.7%) | 534 (44.5%) |
|  |  | Confirmation | 603 | 80.7 (24.5) | 0.0–100.0 | 8 (1.3%) | 279 (46.3%) |
| IWQOL-Lite-CT Physical composite |  | Estimation | 1,197 | 73.2 (22.4) | 0.0–100.0 | 6 (0.5%) | 128 (10.7%) |
|  |  | Confirmation | 602 | 74.8 (21.4) | 3.6 –100.0 | 0 (0.0%) | 68 (11.3%) |
| IWQOL-Lite-CT Physical Function composite |  | Estimation | 1,197 | 73.8 (23.6) | 0.0–100.0 | 10 (0.8%) | 189 (15.8%) |
|  |  | Confirmation | 602 | 75.6 (22.3) | 5.0–100.0 | 0 (0.0%) | 105 (17.4%) |
| IWQOL-Lite-CT Psychosocial composite |  | Estimation | 1,197 | 80.0 (19.5) | 0.0 - 100.0 | 2 (0.2%) | 92 (7.7%) |
|  |  | Confirmation | 602 | 81.5 (18.9) | 3.8–100.0 | 0 (0.0%) | 54 (9.0%) |
| IWQOL-Lite-CT Total Score |  | Estimation | 1,196 | 77.7 (18.8) | 0.0–100.0 | 1 (0.1%) | 53 (4.4%) |
|  |  | Confirmation | 602 | 79.2 (18.2) | 12.5 – 100.0 | 0 (0.0%) | 23 (3.8%) |
| APPADL Score |  | Estimation | 1,200 | 74.7 (21.3) | 0.0–100.0 | 3 (0.3%) | 145 (12.1%) |
|  |  | Confirmation | 603 | 75.0 (21.7) | 3.6–100.0 | 0 (0.0%) | 83 (13.8%) |
| IWQOL-Lite-CT Item 7 |  | Estimation | 1,197 | 1.7 (1.0) | 1.0–5.0 | 747 (62.4%) | 30 (2.5%) |
|  |  | Confirmation | 602 | 1.6 (1.0) | 1.0–5.0 | 384 (63.8%) | 9 (1.5%) |
| IWQOL-Lite-CT Item 20 |  | Estimation | 1,196 | 1.7 (1.1) | 1.0–5.0 | 775 (64.8%) | 57 (4.8%) |
|  |  | Confirmation | 602 | 1.6 (1.0) | 1.0–5.0 | 420 (69.8%) | 19 (3.2%) |
| Weight (KG) |  | Estimation | 1,187 | 85.3 (21.1) | 45.2–219.3 | 0 (0.0%) | 0 (0.0%) |
|  |  | Confirmation | 597 | 84.2 (20.7) | 45.0–178.7 | 0 (0.0%) | 0 (0.0%) |

Abbreviations: APPADL = Ability to Perform Physical Activities of Daily Living; IWQOL-Lite-CT = Impact of Weight on Quality of Life-Lite Clinical Trials Version; IW-SP = Impact of Weight on Self-Perception; SD = standard deviation

**Supplementary Table S2. Responsiveness^a^ of IW-SP by Half MIC, All Anchors, Confirmation Group**

|  | | **< 0.25 MIC** | | **0.25 to <.75 MIC** | | **0.75 to <1.25 MIC** | | **1.25 to <1.75 MIC** | | **1.75 to <2.25 MIC** | | **2.25 to <2.75 MIC** | | **> 2.75 MIC** | | **Overall F-test ^b^** | | **Pairwise comparison ^c^** |
| --- | --- | --- | --- | --- | --- | --- | --- | --- | --- | --- | --- | --- | --- | --- | --- | --- | --- | --- |
| **Anchor** | **Mean Type** | **N** | **Mean** | **N** | **Mean** | **N** | **Mean** | **N** | **Mean** | **N** | **Mean** | **N** | **Mean** | **N** | **Mean** | **F** | **p-value** | **(P-value)** |
| IWQOL-Lite-CT Physical composite MIC | Raw Mean (SD) | 64 | 8.1 (14.1) | 95 | 11.8 (17.8) | 72 | 16.7 (19.8) | 51 | 23.4 (21.0) | 38 | 27.6 (24.6) | 28 | 32.1 (25.6) | 48 | 45.0 (27.1) |  |  | 4**,5**,6***,9*,10**,11***,15*** ,18** |
|  | LS Mean (SE) | 64 | 9.1 (2.6) | 95 | 11.8 (2.1) | 72 | 16.9 (2.4) | 51 | 22.4 (2.9) | 38 | 26.7 (3.3) | 28 | 30.8 (3.9) | 48 | 41.9 (3.1) | 16.22 | <.0001 |  |
| IWQOL-Lite-CT Physical Function composite MIC | Raw Mean (SD) | 67 | 8.5 (15.0) | 126 | 13.8 (16.7) | 34 | 21.1 (23.3) | 68 | 19.5 (18.1) | 26 | 34.0 (26.3) | 35 | 32.9 (25.0) | 47 | 41.5 (30.4) |  |  | 4**,,5***,6***,9*,10**,11***,15* ,18** |
|  | LS Mean (SE) | 67 | 9.1 (2.5) | 126 | 14.2 (1.8) | 34 | 20.9 (3.5) | 68 | 19.5 (2.5) | 26 | 31.9 (4.0) | 35 | 31.2 (3.5) | 47 | 38.2 (3.1) | 14.61 | <.0001 |  |
| IWQOL-Lite-CT Psychosocial composite MIC | Raw Mean (SD) | 109 | 7.0 (13.0) | 93 | 12.2 (14.0) | 59 | 22.3 (20.0) | 33 | 28.8 (18.2) | 24 | 33.7 (18.1) | 24 | 46.9 (23.4) | 43 | 57.8 (23.7) |  |  | 2***,3***,4***,5***,6***,7*,8**,9 ***,10***,11***,14***,15***,17* ,18***,20*** |
|  | LS Mean (SE) | 109 | 6.8 (1.7) | 93 | 12.0 (1.8) | 59 | 22.5 (2.3) | 33 | 29.0 (3.1) | 24 | 33.1 (3.6) | 24 | 46.7 (3.6) | 43 | 57.2 (2.8) | 40.42 | <.0001 |  |
| IWQOL-Lite-CT Total Score MIC | Raw Mean (SD) | 104 | 6.8 (11.4) | 94 | 13.7 (16.4) | 80 | 20.8 (19.5) | 37 | 29.3 (21.2) | 26 | 38.8 (18.6) | 17 | 49.0 (24.1) | 36 | 57.6 (24.7) |  |  | 2***,3***,4***,5***,6***,8**,9*** ,10***,11***,13**,14***,15*** ,17*,18***,20* |
|  | LS Mean (SE) | 104 | 7.0 (1.8) | 94 | 13.6 (1.9) | 80 | 20.7 (2.0) | 37 | 28.8 (3.0) | 26 | 37.9 (3.6) | 17 | 48.6 (4.4) | 36 | 56.8 (3.1) | 34.36 | <.0001 |  |
| APPADL MIC | Raw Mean (SD) | 61 | 9.2 (17.4) | 88 | 14.2 (17.8) | 37 | 17.3 (18.3) | 40 | 22.7 (25.4) | 42 | 23.2 (19.1) | 14 | 26.8 (30.7) | 73 | 40.1 (28.5) |  |  | 6***,11***,15** |
|  | LS Mean (SE) | 61 | 10.3 (2.8) | 88 | 14.5 (2.3) | 37 | 16.6 (3.6) | 40 | 23.3 (3.4) | 42 | 22.6 (3.3) | 14 | 23.0 (5.9) | 73 | 37.2 (2.7) | 11.38 | <.0001 |  |
| Weight change MIC (10%) | Raw Mean (SD) | 39 | 7.5 (10.9) | 129 | 12.7 (18.1) | 129 | 19.1 (23.0) | 88 | 21.8 (22.4) | 51 | 27.0 (25.6) | 20 | 33.3 (26.9) | 12 | 55.6 (26.9) |  |  | 4*,5*,6***,9*,11***,15***,18** ,20* |
|  | LS Mean (SE) | 39 | 8.1 (3.3) | 129 | 12.6 (1.8) | 129 | 19.5 (1.8) | 88 | 21.6 (2.2) | 51 | 25.3 (2.9) | 20 | 30.1 (4.7) | 12 | 52.0 (6.1) | 12.08 | <.0001 |  |
|  |  | **0 Point** | | **1 point** | | **2 points** | | **3 points** | | **4 points** | |  |  |  |  |  |  |  |
| **Anchor** | **Mean Type** | **N** | **Mean** | **N** | **Mean** | **N** | **Mean** | **N** | **Mean** | **N** | **Mean** |  |  |  |  | **F** | **p-value** | **(P-value)** |
| IWQOL-Lite-CT Item 7 ^d,e^ | Raw Mean (SD) | 288 | 9.0 (14.7) | 97 | 25.9 (21.2) | 58 | 40.9 (24.5) | 22 | 48.1 (27.8) | 8 | 64.6 (19.3) |  |  |  |  |  |  | 1***,2***,3***,4***,5***,6***,7** *,9* |
|  | LS Mean (SE) | 288 | 9.3 (1.1) | 97 | 25.3 (1.9) | 58 | 39.6 (2.5) | 22 | 46.4 (4.0) | 8 | 63.0 (6.5) |  |  |  |  | 42.03 | <.0001 |  |
| IWQOL-Lite-CT Item 20 ^d,e^ | Raw Mean (SD) | 293 | 8.5 (13.9) | 91 | 26.9 (19.3) | 43 | 32.9 (22.0) | 34 | 49.0 (25.1) | 18 | 62.0 (27.4) |  |  |  |  |  |  | 1***,2***,3***,4***,6***,7***,8** ,9*** |
|  | LS Mean (SE) | 293 | 8.6 (1.0) | 91 | 26.6 (1.9) | 43 | 32.4 (2.7) | 34 | 48.2 (3.0) | 18 | 60.7 (4.2) |  |  |  |  | 53.47 | <.0001 |  |

Abbreviations: APPADL=Ability to Perform Physical Activities of Daily Living; IWQOL-Lite-CT=Impact of Weight on Quality of Life-Lite Clinical Trials Version; LS=least squares; MIC=Minimal Important Change; SD = standard deviation; SE=standard error

^a^ Baseline to endpoint (Week 40).
^b^ ANCOVA model with covariates of age, gender, and baseline body mass index
^c^ Pairwise comparisons between means were performed using Scheffe's test adjusting for multiple comparisons: 1: < 0.25 MIC vs. 0.25 to <.75 MIC; 2: < 0.25 MIC vs. 0.75 to <1.25 MIC; 3: < 0.25 MIC vs. 1.25 to <1.75 MIC; 4: < 0.25 MIC vs. 1.75 to <2.25 MIC; 5: < 0.25 MIC vs. 2.25 to <2.75 MIC; 6: < 0.25 MIC vs. > 2.75 MIC; 7: 0.25 to <.75 MIC vs. 0.75 to <1.25 MIC; 8: 0.25 to <.75 MIC vs. 1.25 to <1.75 MIC; 9: 0.25 to <.75 MIC vs. 1.75 to <2.25 MIC; 10: 0.25 to <.75 MIC vs. 2.25 to <2.75 MIC; 11: 0.25 to <.75 MIC vs. > 2.75 MIC; 12: 0.75 to <1.25 MIC vs. 1.25 to <1.75 MIC; 13: 0.75 to <1.25 MIC vs. 1.75 to <2.25 MIC; 14: 0.75 to <1.25 MIC vs. 2.25 to <2.75 MIC; 15: 0.75 to <1.25 MIC vs. > 2.75 MIC; 16: 1.25 to <1.75 MIC vs. 1.75 to <2.25 MIC; 17: 1.25 to <1.75 MIC vs. 2.25 to <2.75 MIC; 18: 1.25 to <1.75 MIC vs. > 2.75 MIC; 19: 1.75 to <2.25 MIC vs. 2.25 to <2.75 MIC; 20: 1.75 to <2.25 MIC vs. > 2.75 MIC; 21: 2.25 to <2.75 MIC vs. > 2.75 MIC. *p<0.05; **p<0.01; ***p<0.001.
^d^ IWQOL-Lite-CT individual items will include five groups corresponding to 0 points (no change) and 1, 2, 3, and 4 points of improvement.
^e^ Pairs for IWQOL-Lite-CT individual items: 1: No change vs .1 point improvement, 2: No change vs. 2 points improvement, 3: No change vs. 3 points improvement, 4: No change vs. 4 points improvement, 5: 1 point improvement vs. 2 points improvement, 6: 1 point improvement vs. 3 points improvement, 7: 1 point improvement vs. 4 points improvement, 8: 2 points improvement vs. 3 point improvement, 9: 2 points improvement vs. 4 points improvement, 10: 3 points improvement vs. 4 points improvement.
